# The effect of social support and resource support on emotional exhaustion, insomnia, and suicidal ideation among allied health trainees and post-graduate year doctors in Taiwan

**DOI:** 10.1186/s40359-024-01811-9

**Published:** 2024-06-01

**Authors:** Po-Ching Huang, Chung-Ying Lin, Ru-Yi Huang, Jung-Sheng Chen, Mark D. Griffiths, Carol Strong, Hsiao-Wen Wang, Chiung-Yu Chen, Nai-Ying Ko, Shyh-Jou Shieh

**Affiliations:** 1 School of Physical Therapy, Graduate Institute of Rehabilitation Science, College of Medicine, Chang Gung University, 259, Wen-Hua 1st Rd., Taoyuan, 333323 Taiwan; 2 Biostatistics Consulting Center, College of Medicine, National Cheng Kung University Hospital, National Cheng Kung University, 1, University Rd., East Dist, Tainan, 701401 Taiwan; 3 Department of Public Health, College of Medicine, National Cheng Kung University, 1, University Rd., East Dist, Tainan, 701401 Taiwan; 4 Department of Occupational Therapy, College of Medicine, National Cheng Kung University, 1, University Rd., East Dist, Tainan, 701401 Taiwan; 5 Division of Family Medicine Taipei Tzu Chi Hospital, Buddhist Tzu Chi Medical Foundation, and School of Medicine Tzu Chi University, No. 289, Jianguo Rd., Xindian Dist., New Taipei, 23142 Taiwan; 6 Data Science Degree Program, National Taiwan University and Academia Sinica, No. 1, Sec. 4, Roosevelt Rd., Taipei, 10617 Taiwan; 7 Department of Medical Research, E-Da Hospital, I-Shou University, No.1 Yida Rd., Yanchao Dist, Kaohsiung, 824005 Taiwan; 8 International Gaming Research Unit, Psychology Department, Nottingham Trent University, 50 Shakespeare St, Nottingham, NG1 4FQ UK; 9 Department of Public Health, College of Medicine, National Cheng Kung University, 1, University Rd., East Dist, Tainan, 701401 Taiwan; 10 Department of Hydraulic and Ocean Engineering, College of Engineering, National Cheng Kung University, 1, University Rd., East Dist, Tainan, 701401 Taiwan; 11 Department of Internal Medicine, College of Medicine, National Cheng Kung University Hospital, National Cheng Kung University, 1, University Rd., East Dist, Tainan, 701401 Taiwan; 12 Department of Internal Medicine, College of Medicine, National Cheng Kung University, 1, University Rd., East Dist, Tainan, 701401 Taiwan; 13 Department of Nursing, College of Medicine, National Cheng Kung University, 1, University Rd., East Dist, Tainan, 701401 Taiwan; 14 Department of Surgery, College of Medicine, National Cheng Kung University Hospital, National Cheng Kung University, 1, University Rd., East Dist, Tainan, 701401 Taiwan; 15 Department of Surgery, College of Medicine, National Cheng Kung University, 1, University Rd., East Dist, Tainan, 701401 Taiwan; 16 Institute of Allied Health Sciences, College of Medicine, National Cheng Kung University, 1, University Rd., Tainan, 701401 Taiwan

**Keywords:** COVID-19, Stigma, Support, Insomnia, Suicidality

## Abstract

**Background:**

COVID-19-related stigmatization refers to COVID-19-related judgements by others that devalue the individual. Such stigmatization towards healthcare workers may cause psychological burden and negative consequences. Such stigmatization may have particularly overwhelmed allied health trainees (AHTs) and post-graduate year doctors (PGYDs) because they just started their medical career. Social support and resource support have been reported to benefit psychological health and reduce stigmatization. Therefore, the present study used a cross-sectional study design to investigate the association between perceived stigma, self-stigma, psychological distress, and negative outcomes (including emotional exhaustion, insomnia and suicidal ideation) among AHTs and PGYDs in Taiwan.

**Methods:**

An online survey distributed between July and December, 2022 received 522 responses. Variables were assessed using the 21-item Depression, Anxiety and Stress Scale, Insomnia Severity Index and a series of self-designed questions to assess social support, resource support, perceived stigma, self-stigma, emotional exhaustion, and suicidal ideation.

**Results:**

Structural equation modeling showed that perceived stigma was associated with self-stigma (standardized coefficient [β] = 0.428, *p* < 0.001), and self-stigma was associated with psychological distress (β = 0.197, *p* < 0.001), as well as being associated with emotional exhaustion, insomnia, and suicidal ideation (β = 0.349, 0.556 and 0.212, all *p-*values < 0.001). While social support and resource support were negatively associated with perceived stigma (β= − 0.175 and − 0.152, *p* < 0.01), additional associations were found between social support and emotional exhaustion (β= − 0.093, *p* < 0.001), as well as between resource support and insomnia (β= − 0.120, *p* < 0.001).

**Conclusions:**

The results showed that COVID-19 related stigmatization was correlated to the detrimental consequences of emotional exhaustion, insomnia and suicidal ideation. Clear paths regarding the associations of social support and resource support with the three negative associations were found as the possible solutions. Strategies to reduce the stigmatization and these negative outcomes, or improve the psychological health will benefit AHTs and PGYDs in maintaining a healthy mental status.

## Introduction

In 2019, the highly contagious coronavirus disease 2019 (COVID-19) caused a global disaster [1]. As a novel virus, very little was known about COVID-19 initially [1] and all the clinical interventions or the preventive strategies were based on previous epidemics such as severe acute respiratory syndrome [2]. Subsequently, individuals worldwide were distressed and afraid and may have discriminated against those who were at high-risk of exposure to COVID-19 virus in order to cope with the distress and fear [2]. Frontline healthcare workers and health trainees were among the populations that may have suffered from the COVID-19 stigmatization [2].

Stigmatization is defined as a judgement that devalues the individual [3]. Unfriendly opinions held by others may cause perceived stigma, and internalization by affected individuals can result in self-stigma [3]. Research has shown that stigmatization can be provoked by worldwide or regional pandemics and epidemics [4, 5] and may be experienced by anyone exposed to higher infection risk [6]. Healthcare workers often experience stigma due to the nature of their work [4] which adds extra stress to the lives of an occupational cohort that already experience many stressors not experienced in other professions. A recent study reported that more than 90% of healthcare workers had experienced COVID-19 stigmatization and that half of the them had received hostile attitudes from the general public [7]. Stigmatized individuals can suffer great emotional pressure which can result in psychological distress such as depression and anxiety [8]. Such psychological distress due to stigmatization may further elicit negative psychosocial consequences such as post-traumatic stress disorder, sleep disorders and/or addictive behaviors [6]. Moreover, in the particular context of COVID-19, stigmatized individuals may stay away from their families to avoid unnecessary transmission risk [7], resulting in emotional isolation and further exacerbating the psychological impact [9].

Furthermore, the psychological distress has been associated with consequential effects such as emotional exhaustion [10], insomnia [11], and (in extreme cases) suicidality [12]. As the initial and major component of burnout [13], emotional exhaustion represents prolonged emotional overstress [13] and was commonly found among healthcare workers during the COVID-19 pandemic [14]. It may affect their morale and productivity, leading to a reduction in care quality and an increase the possibility of medical errors [15]. In addition, the time exposed to COVID-19 among healthcare workers has been associated with psychological distress and insomnia [11, 16], and the level of insomnia has been associated with suicidal ideation among medical workers [16].

In Taiwan, students who majored in medicine or allied health-related subjects (e.g., occupational therapy, physiotherapy, nursing, and pharmacy) are required to undertake at least one year of clinical training [17]. These allied health trainees (AHTs) and post-graduate year doctors (PGYDs) are required to practice their clinical technique and learn to be a “real” healthcare worker in clinical settings of teaching hospitals [17]. During the COVID-19 pandemic, their prolonged stay in the hospitals for clinical training may have caused public stigmatization towards them [18, 19]. Moreover, being junior members of the medical industry, AHTs and PGYDs can be overwhelmed by the clinical learning processes and requirements, subsequently causing detrimental impacts on their psychological health and unwanted negative outcomes (e.g., emotional exhaustion, insomnia, and suicidal ideation) [18, 19].

In response to these negative consequences, previous research has examined potential protective and risk factors for mental health problems among AHTs and PGYDs. This has shown associations of social support and resource support in relation to stigmatization and psychological distress [18, 20–23]. Studies have reported that greater social support was associated with better mental well-being during the COVID-19 pandemic [21, 23] and that the interaction between different forms of social support (e.g., from family or work) may work synergically as a psychological support system [22]. Moreover, one study found that stigmatization and psychological distress were likely to worsen due to lack of personal protection equipment or financial hardship [20]. For example, a medical intern from the Democratic Republic of Congo indicated that medical staff should be well-equipped with personal protective equipment because he became stigmatized after treating a suspicious COVID-19 case [18] and consequently suffered severe emotional burden.

**Fig. 1:**
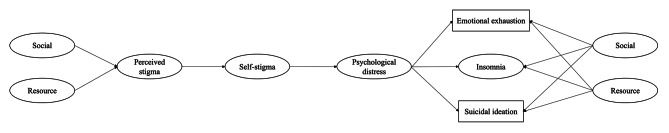
Proposed model explaining the association of support and resource with insomnia, emotional exhaustion and suicidal ideation during the COVID-19 pandemic among healthcare students under going an internship. Social = social support, resource = resource support

Therefore, social support and resource support may have potentially beneficial effects in reducing stigmatization and psychological distress. However, no previous study has examined their joint effect among healthcare workers. Therefore, the purpose of the present study was to investigate the associations of social support and resource support in relation to emotional exhaustion, insomnia, and suicidal ideation among AHTs and PGYDs in Taiwan. Two forms of stigma (i.e., perceived stigma and self-stigma), as well as psychological distress were investigated. The proposed model is outlined in Fig. 1. It was hypothesized that (i) perceived stigmatization would be positively associated with self-stigma and psychological distress; (ii) psychological distress would be positively associated with emotional exhaustion, insomnia, and suicidal ideation; and (iii) social support and resource support would be negatively associated with perceived stigma. It was also hypothesized that (iv) social support and resource support would be negatively associated with these detrimental outcomes.

## Methods

### Participants and procedure

The present study was an exploratory cross-sectional study conducted using convenience sampling and snowball sampling methods. AHTs and PGYDs who met the following inclusion criteria were eligible for participation: (i) aged over 20 years, (ii) studying in any medicine- or allied-health-related fields, (iii) having received clinical training between 2021 and 2022, and (iv) being able to read Chinese. The online survey created using *SurveyMonkey* took place from July to December, 2022. The authors contacted four hospitals and 10 university departments/units in Taiwan to help distribute the survey link to potential participants. An e-consent form was included in the first page of the online survey. By clicking “yes”, the participants gave their informed consent to participate. When clicking “no”, the survey could not be accessed. In addition, participants were encouraged to disseminate the survey link to others who met the criteria. In order to confirm the participants’ eligibility, three questions were asked for verification purposes. The first question asked if individuals majored in medical specialties. Those who indicated that they majored in non-medical fields were unable to access the survey. The second question asked about their specific major. All the medical specialties which involved on-site hospital training were listed and an option of “none of the above”. Individuals who chose this latter option were unable to access the survey because they may not be a major in medical specialties or were in specialties that did not require clinical training in hospitals. The last question asked individuals what year they received their clinical training. If the answer was not within the survey period (i.e., between 2021 and 2022), they were unable to access the online survey. The study protocol was approved by the National Cheng Kung University Human Research Ethics Committee with the approval number of NCKU HREC-E-111-325-2.

### Measures

Participants’ demographics including age, gender, and major subject of study were recorded for background assessment. Independent variables including social support, resource support, perceived stigma, self-stigma and psychological distress, as well as the outcome variables of emotional exhaustion, insomnia and suicidal ideation were assessed using specific items or questionaries.

*Social support* was defined as the provision of assistance that comforts the individual [24]. It may derive from the individuals’ families or work colleagues. Three items rated on a five-point Likert scale (1 = strongly disagree, 5 = strongly agree) were adapted from a previous study [25]. The scores were averaged and a higher score indicates a higher level of perceived social support. A sample item was “*In the past week, my family has given me enough support*”. The items demonstrated acceptable internal consistency in the present study (Cronbach’s α = 0.78).

*Resource support* was defined as resources that helped individuals cope with the COVID-19 pandemic. Five items including personal protective supplies (e.g., mask, gloves or alcohol sanitizer), knowledge or information regarding the COVID-19, medical support, psychological support and money were developed based on a previous study [26] and assessed using a three-point Likert scale (1 = extremely insufficient, 3 = extremely sufficient). The scores were averaged and a higher score indicated the individual possessed sufficient supplies to cope with COVID-19. A sample item was “*I have enough money to get through the COVID-19 pandemic*”. The items demonstrated acceptable internal consistency in the present study (α = 0.769).

*Perceived stigma* was defined as the external judgement derived from COVID-19 pandemic that devalues the individual [3]. Seven yes-no questions (yes = 1, no = 0) were adapted from the Perceived Weight Stigma Scale [27, 28]. The scores were summed to generate the total scores ranging from 0 to 7. A higher score indicated a higher level of perceived stigmatization. A sample item was “*I have been stigmatized in social situations (e.g., workplace)*”. The items demonstrated very good internal consistency in the present study (α = 0.869).

*Self-stigma* was defined as the internal devaluation derived from the unfriendly self-evaluation because of COVID-19 pandemic [3]. Nine items rated on a four-point Likert scale (1 = strongly disagree, 4 = strongly agree) were adapted from the Self-Stigma Scale-Short version [28, 29]. The scores were averaged and a higher score indicated a higher level of self-stigmatization derived from COVID-19. A sample item was “*I avoid social interaction because I may have the COVID-19 infection*”. The items demonstrated excellent internal consistency in the present study (α = 0.900).

*Psychological distress* was assessed using the 21-item Depression, Anxiety and Stress Scale (DASS-21). Items were rated on a four-point Likert scale (0 = never, 3 = almost always). In the present study, the scores of the total 21 items were summed and multiplied by 2 to generate the overall DASS-21 scores ranging from 0 to 126 [30]. A higher score indicated a higher level of psychological distress. The psychometric properties of the Chinese version of DASS-21 have been found satisfactory in prior research [31–34]. A sample item was “*I found it hard to wind down*”. The scale demonstrated excellent internal consistency in the present study (α = 0.950).

*Emotional exhaustion* was defined as individuals being overwhelmed and drained to the extent that they are not able to meet their required daily demands [35]. One item rated on a seven-point Likert scale (1 = strongly disagree, 7 = strongly agree) was adapted from the Emotional Exhaustion Scale [36] to assess the emotional exhaustion derived from COVID-19. A higher score indicated a higher level of being emotionally worn-out. The item was “*My academic learning has been affected due to the COVID-19 pandemic because it has made me feel tense and drained, even a sense of exhaustion right after I wake up.*”

*Insomnia* was assessed using Insomnia Severity Index (ISI). The ISI contains seven items rated on a five-point Likert scale (0 = none / not disturbing at all, 4 = very severe/extremely disturbing). The scores were summed and total scores ranged from 0 to 28. A higher score indicated a higher level of insomnia. A sample item was *“How SATISFIED/DISSATISFIED are you with your CURRENT sleep pattern?”.* The psychometric properties of the Chinese version ISI have been found satisfactory in prior research [37]. The scale demonstrated very good internal consistency in the present study (α = 0.893).

*Suicidal ideation* was defined as thoughts and ideas about taking one’s own life because of COVID-19 [35]. One item rated on a five-point Likert scale (1 = never, 5 = every day) was adapted from two previous studies [25, 38]. A higher score indicated a higher level of suicidal ideation. The item was “*I have thought about suicide because of COVID-19 in the past week*”.

### Statistical analysis

All the study variables were checked for normality using skewness and kurtosis. Participants’ characteristics were summarized using descriptive analysis and the correlations between the studied variables were calculated using Pearson’s correlation coefficient. Structural equation modeling (SEM) with the maximum likelihood as the estimator was used to examine the fitness of the proposed model. Four fit indices [39] including comparative fit index (CFI), Tucker-Lewis index (TLI), root mean square error of approximation (RMSEA), and standardized root mean squared residual (SRMR) were used to examine support for the model fit. The suggested level of CFI and TLI needs to be higher than 0.9 and the level of RMSEA and SRMR needs to be lower than 0.08 [39]. In addition, the mediating effect of perceived stigma, self-stigma, and psychological distress were further examined using the default setting of 1000 bootstrapping resamples. The *lavaan* package in R software and SPSS 26.0 (IBM, Armonk, New York) were used to perform SEM and the rest of the statistical analysis. The significance level was set at *p* < 0.05 (two-tailed test).

## Results

A total of 853 participants gave their consent to participate. However, 45 were excluded because of non-medicine- or non-allied-health-related specialties, and 286 were excluded because the year they received clinical training fell outside of that required in the inclusion criteria. Therefore, a total of 522 participants met the eligibility criteria and their responses were processed for analysis. The results of normality showed that almost all of the variables demonstrated the absolute values of skewness ranged between 0.112 and 1.613 and kurtosis ranged between 0.336 and 1.673, except for suicidal ideation (skewness = 3.290 and kurtosis = 11.340). Despite the relatively high values, these values did not exceed the problematic limit of 20 [40, 41]. Therefore, statistical analysis was conducted following the assumption of normal distribution.

Table 1 shows the participants’ characteristics. Most of the participants were female (*n* = 334; 63.98%) with the mean age of 24.5 years old. The participants mostly majored in medicine (*n* = 195; 37.36%), nursing (*n* = 101; 19.35%) and pharmacy (*n* = 67; 12.84%), followed by occupational therapy (*n* = 65; 12.45%), physical therapy (*n* = 51; 9.77%), medical science and biotechnology (*n* = 7; 1.34%) and social work (*n* = 3; 0.57%). Additionally, 33 participants (6.32%) responded “other” and majored in respiratory therapy (*n* = 22; 4.21%), Chinese medicine (*n* = 6; 1.15%), dentistry (*n* = 3; 0.57%), and speech therapy (*n* = 2; 0.38%).

**Table 1:** Participants’ characteristics (*n* = 522)

	Mean (SD) or *n* (%)
Age	24.5 (3.6)
Gender (female)	334 (63.98)
Study major	
Medicine	195 (37.36)
Nursing	101 (19.35)
Pharmacology	67 (12.84)
Social work	3 (0.57)
Occupational therapy	65 (12.45)
Physical therapy	51 (9.77)
Medical science and biotechnology	7 (1.34)
Other	33 (6.32)
Social support (range 1–5)	4.08 (0.64)
Resource support (range 1–3)	2.68 (0.41)
Perceived stigma (range 0–6)	1.10 (1.85)
Self-stigma (range 1–4)	2.17 (0.68)
Psychological distress (range 0-126)	18.92 (21.21)
Emotional exhaustion (range 1–7)	3.63 (1.50)
Insomnia (range 0–28)	6.92 (5.00)
Suicidal ideation (range 1–5)	1.26 (0.72)

The correlations between the studied variables are shown in Table 2. All the studied variables were significantly correlated with each other (*r*-values = -0.290 to 0.583), except for the correlation between resource support and suicidal ideation.

**Table 2:** Correlation between studied variables (*n* = 522)

	1	2	3	4	5	6	7	8
^1^ Social support	–							
^2^ Resource support	**0.173** **(< 0.001)**	–						
^3^ Perceived stigma	**–0.181** **(< 0.001)**	**–0.113** **(0.010)**	–					
^4^ Self-stigma	**–0.199** **(< 0.001)**	**–0.163** **(< 0.001)**	**0.432** **(< 0.001)**	–				
^5^ Psychological distress	**–0.290** **(< 0.001)**	**0.340** **(< 0.001)**	**0.153** **(< 0.001)**	**0.243** **(< 0.001)**	–			
^6^ Emotional exhaustion	**–0.180** **(< 0.001)**	**–0.119** **(0.007)**	**0.186** **(< 0.001)**	**0.353** **(< 0.001)**	**0.361** **(< 0.001)**	–		
^7^ Insomnia	**–0.217** **(< 0.001)**	**–0.261** **(< 0.001)**	**0.220** **(< 0.001)**	**0.305** **(< 0.001)**	**0.583** **(< 0.001)**	**0.374** **(< 0.001)**		
^8^ Suicidal ideation	**0.100** **(0.023)**	–0.022 (0.612)	**0.216** **(< 0.001)**	**0.074** **(0.090)**	**0.218** **(< 0.001)**	**0.162** **(< 0.001)**	**0.109** **(0.012)**	–

Significant correlations are shown in **bold**

**Fig. 2:**
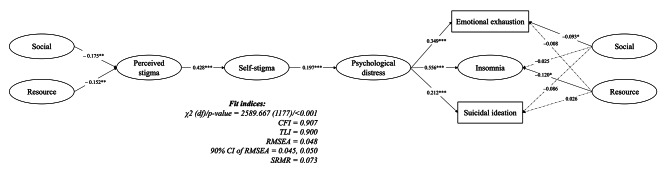
Results of structural equation modeling examining the proposed model (*n* = 522). Solid line indicates a significant effect; dashed line indicates a non-significant effect. *p < 0.05; **p < 0.01; ***p < 0.001. Social = social support, resource = resource support

The SEM results (Fig. 2) demonstrated a well-fitting model with supportive fit indices (CFI = 0.907, TLI = 0.900, RMSEA = 0.048 and SRMR = 0.073). More specifically, social support and resource support were negatively correlated with perceived stigma (standardized coefficient [β] = -0.175, *p* = 0.004 and − 0.152, *p* = 0.031). Perceived stigma was positively correlated with self-stigma (β = 0.428, *p* < 0.001), and self-stigma was positively correlated with psychological distress (β = 0.197, *p* < 0.001). Significant associations were found between psychological distress and the three outcome variables of emotional exhaustion, insomnia, and suicidal ideation (β = 0.349, 0.556 and 0.212, all *p*-values < 0.001). Moreover, significant negative correlations were found between (i) social support and emotional exhaustion (β = -0.093, *p* = 0.045), and (ii) resource support and insomnia (β = -0.120, *p* = 0.014).

The mediation effects of perceived stigma, self-stigma along with psychological distress are shown in Table 3. In brief, perceived stigma, self-stigma, and psychological distress significantly mediated all the associations. More specifically, the significant mediation effects were found in the association of social support to emotional exhaustion, insomnia, and suicidal ideation (β = − 0.008, − 0.003 and − 0.005), and in the association of resource support to the three negative outcomes (β = − 0.007, − 0.003 and − 0.005).

**Table 3:** Mediation effect in the proposed model

	Independent variable	Mediator 1	Mediator 2	Mediator 3	Outcome variable	Standardized coefficient	95% bootstrapping confidence interval	
							Lower limit	Upper limit
1	Social	Perceived stigma	Self-stigma	Psychological distress	Emotional exhaustion	**-0.008**	-0.018	-0.002
2					Insomnia	**-0.003**	-0.007	-0.001
3					Suicidal ideation	**-0.005**	-0.011	-0.001
7	Resource				Emotional exhaustion	**-0.007**	-0.018	-0.001
8					Insomnia	**-0.003**	-0.007	-0.000
9					Suicidal ideation	**-0.004**	-0.011	-0.001

Significant correlations are shown in **bold**. Social = social support, resource = resource support

## Discussion

The present study investigated the effects of social support and resource support affecting emotional exhaustion, insomnia, and suicidal ideation among allied health trainees (AHTs) and post-graduate year doctors (PGYDs). The results showed that perceived stigma was found among the studied population and was associated with self-stigma. Self-stigma was associated with psychological distress, which was in turn associated with the negative outcomes of emotional exhaustion, insomnia and suicidal ideation. However, both social support and resource support were negatively associated with perceived stigma. Social support was associated with emotional exhaustion and resource support was associated with insomnia. The present study also examined the mediation effects via the perceived stigma, self-stigma, and psychological distress. The findings demonstrated that sequential mediation effects were found in all the associations of social support and resource support for the three negative outcomes. These findings demonstrated that perceived stigma, self-stigma, and psychological distress due to COVID-19 can result in detrimental consequences (i.e., emotional exhaustion, insomnia and suicidal ideation) among AHTs and PGYDs, which have been reported to affect the quality of medical care [9]. However, social support and resource support can negatively affect these unwanted impacts. Therefore, the influences of both social support and resource support should be further investigated because these individuals are going to be the future healthcare providers.

The present study showed that perceived stigma was associated with self-stigma, which was in turn associated with psychological distress. Terror management theory [42] suggests that uncertainty triggers the defense response which results in individuals targeting on things that are considered as the threat to life. Similarly, labeling theory [43] proposes that stigmatization provides a sense of relief when labeling uncertainty [44] because stigmatizing others can be considered as a strategy to avoid the danger and protect the community, which generates psychological comfort [45]. Therefore, the uncertainty of COVID-19 could have caused the public to have stigmatizing thoughts about healthcare workers because medical work contains the risk of infection [44]. Individuals being stigmatized may have to accept such judgement and labels, and internalize the stigmatization, causing self-stigma and resulting in psychological distress [3].

Additionally, in the present study, psychological distress was associated with negative outcomes including emotional exhaustion, insomnia, and suicidal ideation. The associations between psychological distress and such negative outcomes have been widely reported previously [10–12]. More specifically, these negative outcomes may mutually interact. Studies have shown that as the major component of burnout, emotional exhaustion can significantly worsen insomnia [46]. In addition, the severity of insomnia [47], and burnout [13] were highly associated with suicidality, especially when individuals were depressed [47]. Therefore, strategies to reduce the psychological distress or alleviate these negative outcomes can be implemented among AHTS and PGYDs to improve their quality of life. For example, mindfulness therapy [48] and interventions to improve the sleep quality [49] can be used to reduce psychological distress as well as insomnia.

Both social support and resource support were negatively associated with perceived stigma in the present study. Social support is considered as one of the effective ways in reducing stigmatization [50] because it provides a safe place for stigmatized individuals to express, share, and discuss possible solutions with each other, creating a supportive network to empower those within them [50]. However, only a few healthcare workers receive sufficient social support [21], and low social support was reported to be a risk factor for psychological distress, sleep problems, and suicidal thoughts in a previous study [51]. Another study showed that lower self-esteem, higher depression and anxiety, as well as poor sleep quality were associated with stigmatization, but these outcomes were totally reversed when individuals had social support [52]. Medical students with social support have also been reported to have higher empathy and self-efficacy [53], suggesting the importance of social support among healthcare workers [53].

On the other hand, resource support included personal protective equipment (e.g., mask, gloves, alcohol sanitizer), knowledge or information, medical support, psychological support, and money. These resources may independently or interactively demonstrate their impact on stigmatization. For example, Yufika et al. [54] reported that doctors experience less stigmatization which may result from their higher knowledge level when compared to other healthcare workers. They also found that healthcare workers without COVID-19-related training experienced less stigma which may because of knowing the infectious risk of COVID-19, but lacking sufficient personal protective equipment may exacerbate the stigma. These results support the effect of resource support on stigmatization and was corroborated by the findings of the present study. In addition, the present study’s findings regarding the negative associations between social support and emotional exhaustion, as well as resource support and insomnia, have been reported in a previous study [55]. Suggested approaches, such as hosting support groups for healthcare workers or providing them with educational awareness training regarding the COVID-19, can be taken into consideration by hospitals and government stakeholders in order to maintain the healthy mental status of healthcare workers.

The reason why AHTs and PGYDs may be more vulnerable to public’s stigmatization is because that they may lack the experience to deal with the stigmatized situation. One study reported that service duration of frontline government workers with pandemic control duty acted as a protective factor for insomnia due to their greater experience in stressful situations [6]. In addition, close relatives may put stress on the AHTs and PGYDs by worrying about the uncertainty and risk of their job duties, which could also cause a double stigmatization [2, 19]. Indeed, the present study’s findings may (at least in part) be attributed to the context of the COVID-19 pandemic and its impact on AHTs and PGYDs because the infectious disease may result in higher stigma towards medical workers [4, 56] and medical trainees have little or no experience in dealing with stigmatization. According to the findings, providing sufficient social support and resource support can reduce the stigma level among healthcare workers. It has also been suggested that a clear explanation on the job role to the relatives can reduce excessive worries [19]. In addition, anti-stigma strategies, such as improving public awareness or reducing the misunderstanding toward the medical workers such as medical workers carrying virus or having a suspected infection [57], should always be adopted irrespective of whether there is a pandemic.

The present study has several limitations. First, the use of self-report surveys may result in social desirability bias (i.e., the participants may give the response that tends more to social expectation) or recall bias (i.e., the participants may not be able to accurately recall their experiences). Second, the cross-sectional study design used in the present study prevents knowing the cause-and-effect relationship between the studied variables. Third, the relatively small sample size may lack representativeness regarding the target population and therefore limit the generalizability of the findings. Fourth, the studied population of AHTs and PGYDs may lack generalizability to all medical workers. Compared to senior medical workers, AHTs and PGYDs with relatively less work experience may exaggerate their psychological distress, which further affects the implementation of the study’s findings. Fifth, the study did not formally assess the specific contribution of each social support or resource support, therefore there is no information regarding the particular effect of specific support. Sixth, the data regarding individuals’ clinical training (e.g., the duration for clinical training, the timing in starting the internship, and the contact level with patients) was not collected. Such factors would likely have had an influence on the study’s findings. Seventh, the COVID-19 pandemic, as well as the factors such as friends, family or workplace can be the confounders that affect the stigma and negative outcomes, and were not controlled for in the present study. Further study is needed to confirm if the present study’s findings still hold after controlling for these variables. It has also consistently been shown that medical training can (in and of itself) result in many negative psychosocial consequences (e.g., severe stress, sleep disorders, eating disorders, alcohol abuse, suicide behaviors) [18, 19]. This is a confounding variable which could have (in part) contributed towards the findings. However, this was not controlled for in the present study. Further study is needed to confirm if the present study’s findings still hold after controlling for being a medical trainee. Eighth, although the authors contacted several departments/units and hospitals in Taiwan, some institutions or departments may have been more represented than others, which may have resulted in sampling bias.

Despite these limitations, the present study provided empirical evidence regarding the seemingly beneficial effects of social support and resource support in reducing stigmatization among AHTs and PGYDs, which if not addressed may further be associated with psychological distress and the consequential negative outcomes. Considering that worldwide or regional pandemic/epidemics are likely to happen in the future and have occurred in the recent past (for example, there was only a 16-year gap between the SARS epidemic in 2003 and the COVID-19 pandemic in 2019), the present findings provide suggestions and guidance for future preparedness to prevent any further negative consequences for healthcare workers. According to the results, self-help intervention, as well as strategies such as mindfulness [48] or family education [19], can be taught to healthcare workers to alleviate the impact of stigmatization and reduce the psychological distress. For stakeholders and hospitals, providing sufficient social support (e.g., hosting support group for AHTs and PGYDs) and resource support (e.g., providing essential protective equipment) may benefit healthcare workers. In addition, COVID-19-related information can be disseminated to the public to improve the knowledge of COVID-19 and reduce the stigmatization towards healthcare workers. These approaches would likely contribute to a more friendly environment to support healthcare workers, especially AHTs and PGYDs.

## Conclusions

The present study investigated the effects of social support and resource support on the negative outcomes of emotional exhaustion, insomnia and suicidal ideation while also simultaneously studying perceived stigma, self-stigma, and psychological distress among Taiwanese AHTs and PGYDs. The results demonstrated the significant associations of social support and resource support with perceived stigma, which was associated with self-stigma. Self-stigma was further associated with psychological distress that was also associated with the negative outcomes of emotional exhaustion, insomnia, and suicidal ideation. In addition, social support was associated with emotional exhaustion, and resource support was associated with insomnia. Strategies including mindfulness, public education to increase knowledge or interventions to improve sleep quality, as well as social support given by next-of-kin or peers and resource support provided by the hospitals or government stakeholders, are potential actions to help reduce the psychological burden and unwanted negative consequences among healthcare workers.

## Data Availability

The datasets used and/or analyzed during the current study are available from the corresponding author on reasonable request.

## Abbreviations

COVID-19: Coronavirus disease 2019
AHTs: Allied health trainees
PGYDs: Post-graduate year doctors
DASS-21: Depression, Anxiety and Stress Scale
ISI: Insomnia Severity Index
SEM: Structural equation modeling
CFI: Comparative fit index
TLI: Tucker-Lewis index
RMSEA: Root mean square error of approximation
SRMR: Standardized root mean squared residual
